# Modulation of T-cell responses by anti-tumor necrosis factor treatments in rheumatoid arthritis: a review

**DOI:** 10.1186/s13075-018-1725-6

**Published:** 2018-10-12

**Authors:** Jean-Luc Davignon, Benjamin Rauwel, Yannick Degboé, Arnaud Constantin, Jean-Fredéric Boyer, Andrey Kruglov, Alain Cantagrel

**Affiliations:** 1 Centre de Physiopathologie Toulouse Purpan, INSERM-CNRS-UPS, UMR 1043, CHU Purpan, 1 Place Baylac, 31024 Toulouse Cedex, France; 2 Centre de Rhumatologie, CHU de Toulouse, 31059 Toulouse, France; 3 Faculté de Médecine, Université Paul Sabatier Toulouse III, 31062 Toulouse, France; 4 Lomonosov Moscow State University, 119991 Moscow, Russia; 5 German Rheumatism Research Center (DRFZ), 10117 Berlin, Germany

**Keywords:** Rheumatoid arthritis, Anti-TNF, Biotherapy, T-cell

## Abstract

Tumor necrosis factor (TNF) is a pleiotropic cytokine involved in many aspects of immune regulation. Anti-TNF biological therapy has been considered a breakthrough in the treatment of chronic autoimmune diseases, such as rheumatoid arthritis (RA). In this review, because of the major involvement of T cells in RA pathogenesis, we discuss the effects of anti-TNF biotherapy on T-cell responses in RA patients. We also outline the potential fields for future research in the area of anti-TNF therapy in RA.

This could be useful to better understand the therapeutic efficiency and the side effects that are encountered in RA patients. Better targeting of T cells in RA could help set more specific anti-TNF strategies and develop prediction tools for response.

## Background

The discovery of the role of tumor necrosis factor (TNF) in the pathogenesis of rheumatoid arthritis (RA) has led to anti-TNF biological therapy as a breakthrough in the treatment of chronic autoimmune diseases, such as RA, Crohn’s disease, psoriatic arthritis, and spondyloarthritis [1]. Various anti-TNFs are currently used for the treatment of RA, including infliximab (IFX), a chimeric antibody, and two fully human antibodies adalimumab (ADA) and golimumab. Additionally, etanercept (ETA) is a human recombinant dimeric fusion protein consisting of two soluble p75 TNF-RII chains linked to a modified Fc portion of human IgG. Finally, certolizumab pegol (CZP) is a pegylated Fab’ fragment of a humanized anti-TNF antibody. Biosimilars of IFX and of ETA are already in use.

TNF is a pleiotropic cytokine involved in many aspects of immune regulation [2]. TNF is first synthesized as a biologically active transmembrane homotrimer (tmTNF), which is further released upon cleavage by tumor necrosis factor-alpha converting enzyme (TACE, also named ADAM17) protease. Soluble TNF binds to the receptors TNF-RI and TNF-RII, while tmTNF binds preferentially to TNF-RII. Anti-TNF biologics can block both soluble and tmTNF [3]. TNF can be produced by multiple cell types such as T and B cells and innate immune cells (dendritic cells, monocytes, neutrophils, mast cells). All these sources may contribute to the development of a pathological state of chronic inflammation, especially in RA. T cells are also targets of TNF either directly, like all cells that express TNF-Rs, or indirectly as a result of antigen presentation or costimulation. The immunomodulatory role of TNF-R2 on T-cell activity has been described in the collagen-induced arthritis (CIA) model of arthritis [4].

In this review, because of the major involvement of T cells in RA pathogenesis, we discuss the effects of anti-TNF biotherapy on T-cell responses in RA patients. This could be of help for the interpretation of the clinical effects (or lack thereof) of anti-TNF treatments, as well as being useful to better understand the side effects which are encountered in RA patients.

## Role of T cells in RA

Much has been learned from mouse models in the understanding of RA, especially regarding the role of T cells. Collagen-induced, K/BxN, IL-1 RA-KO, and SKG models were shown to depend on T lymphocytes [5]. More specifically, the SKG model, depending on a mutation in ZAP 70 that affects the TcR-ζ chain signaling and T-cell selection, directly implicated the role of T cells in the development of experimental arthritis.

There has been an ongoing debate over the respective importance of macrophages and T cells. The presence of T cells in joints and the expansion of clonotypic T cells, as a result or a cause of inflammation, in the synovium of RA patients has fueled that debate. The role of the HLA-DR shared epitope in the development of RA is a strong indication for the role of T cells [6]. There is a T-cell response to citrullinated T-cell epitopes or PAD peptides [7] in patients who bear the RA susceptibility HLA-DR allele. A direct argument for the role of T lymphocytes in RA has been the successful use of CTLA4-Ig as a biotherapy that blocks the CD28-CD86/CD80 interaction [8]. Thus, the current view is that there is an interplay between pathogenic T cells, macrophages, and cytokines that contributes to the pathogenic imbalance in RA [9] and can be targeted with biologics.

## Role of TNF in the development of the immune system

TNF has been shown to be essential in many stages of T-cell development. In the thymus, TNF promotes the apoptosis of triple-negative CD3/CD4/CD8 [10] and double positive CD4/CD8 thymocytes [11], as well as the development of single positive thymocytes [12]. Thus, it is expected that treatment of infants with anti-TNF might alter the development of their T cells. This needs further investigation.

Secondary lymphoid organs (SLO) are crucial for the development of efficient adaptive immune responses. Organized in well-demarcated T-cell zones and B-cell follicles, SLO bring the antigen that is trapped by various subsets of dendritic cells (DCs) in close contact with the immune cells, provide costimulatory signals from DCs, and thereby initiate an appropriate immune response.

TNF-mediated signaling is crucial for the development of some and for structural maintenance of most of the SLO. Distinct cellular sources and molecular forms of TNF contribute to the organization of SLO microarchitecture. TNF from B and T cells cooperates to maintain the structural integrity in lymph nodes, which are indispensable for the generation of efficient local immune responses.

The requirement of TNF signaling for organized lymphoid structures in mice was confirmed by studies in humans. Rheumatoid arthritis patients receiving ETA lack germinal center development in their tonsils [13]. Similar experiments in mice showed that pharmacological inhibition of TNF by ETA leads to inhibition of follicular dendritic cell development and a subsequent decrease in germinal center response, as well as a reduction in the marginal zone [14]. However, the structure of B-cell follicles in the spleen remained unchanged, suggesting that some of the TNF-dependent features of splenic microarchitecture are not inhibited by ETA [14].

Altogether, TNF controls the development and organization of SLO structures and, thereby, influences the development of adaptive immune responses. This could be of importance during the follow-up of RA patients, especially children, treated with anti-TNF.

## Role of TNF in T-cell differentiation, activation, and maturation: action of TNF inhibitors

Activation of naive T cells is initiated during their encounter with antigen peptide presented by mature DCs. This activation is dependent on coactivation mediated by the membrane interaction between members of the TNF/TNF-R family other than TNF cytokine on T cells and DCs. As a cytokine, TNF contributes to efficient antigen presentation by inducing DC maturation.

Interaction of T cells with antigen presenting cells leads to differentiation into effector and memory T cells (reviewed in [15]). To understand how anti-TNF treatment may exert an impact on the pathogenicity of T lymphocytes, we first need to overview the role of TNF in the activation of effector, memory, and regulatory T cells.

TNF is reported to negatively regulate the expansion of effector CD4^+^ and CD8^+^ T cells during viral infection through apoptosis, thus subsequently limiting the T cell memory compartment [16]. TNF, acting along with interleukin (IL)-33, transforming growth factor (TGF)-β and IL-15, induces resident memory T cells (T_RM_) with CD69 and CD103 expression [15]. These T cells do not recirculate and remain in the lymphoid tissue. Production of TNF by T_RM_ in turn contributes to the maturation of DCs and efficient Ag presentation for recall T-cell activation. Anti-TNF biologics are thus expected to modulate the effector and the memory T-cell response during infections and vaccination (vide infra).

To invade inflamed tissue, T lymphocytes must have the capacity to traffic through endothelial cell junctions. This phenomenon, called diapedesis, has been shown to depend on TNF and interferon (IFN)-γ [17]. Thus, although this has never been tested formally, anti-TNF drugs have the capacity to reduce inflammation by interfering with diapedesis and migration of T cells to the joints.

However, TNF has a contrasting role in T-cell activation [18, 19]. The notion of long-term pathogenic effects of TNF in disease was pioneered by Maini and Feldmann, based on the observation of elevated TNF production in the joints of RA patients. They also reported that chronic exposure of cells to TNF impaired the T cell-specific recall response to tetanus toxoid. This inhibition was later shown to be due to attenuation of TcR signaling. At the molecular level, TNF appeared to inhibit CD3-ζ chain expression via Src-like adaptor protein (SLAP) degradation [20].

Toxicity of IFX for T cells is minimal and the metabolism of T cells is not significantly altered by anti-TNF [21]. However, T-cell subsets were not investigated, and this requires further studies. Regarding in-vitro T-cell activation, impairment of T cells from RA patients can be reversed by anti-TNF and, correspondingly, anti-TNF treatment of RA patients restores in-vitro proliferation in response to soluble antigens [22]. In a model of transmembrane expression of TNF in the Jurkat T-cell line (tm-Jurkat), Mitoma et al. [23] showed that IFX induces JNK activation and IL-10 production, and inhibits proliferation. Reverse signaling is a mechanism of signaling mediated by anti-TNF or TNF-R through binding to tmTNF [3]. Reverse signaling has been suggested to regulate inflammation in macrophages and T cells. However, the molecular mechanisms are not completely understood and demonstration of in-vivo reverse signaling has yet to be demonstrated.

Another possible mechanism of action of anti-TNF on T cells is the regulation of cell death. The action of anti-TNF drugs on cell death was tested using the Jurkat T-cell line transfected with tmTNF [24]. Due to the absence of the Fc fragment, CZP did not induce antibody-dependent cell-mediated cytotoxicity or complement-dependent cytotoxicity, whereas golimumab, IFX, and ADA did. CZP and ETA did not induce apoptosis in tmTNF Jurkat cells [24]. However, those data were obtained with cells overexpressing tmTNF and cannot be extrapolated to physiologic conditions. In tm-Jurkat T-cells, ADCC and CDC, induced by IFX and ADA, were of lower intensity than with ETA, and were not observed with CZP [24].

In patients with active RA, spontaneous apoptosis of CD4^+^CD25^+^ cells was evaluated at the start of treatment with IFX and after 3 months of treatment [25]. It was shown that spontaneous in-vitro apoptosis of CD4^+^CD25^+^ cells, which was increased in RA patients compared with healthy donors, was reduced after treatment with IFX [25].

## Effects of anti-TNF on T-helper cell subset differentiation

There is now a growing literature in RA patients on increased T helper (Th)1 [26–29] and Th17 [26, 27, 29, 30] responses following TNF blockade. Th17 and shifting to nonclassic Th1 have been described as potential components of the pathophysiology of RA, but their overall significance is debated [31].

Hull et al. reported that patients responding to ADA or ETA had an increase in circulating Th17 [30]. Conversely, an increase in Th17 has been reported in patients not responding to TNF inhibitors [27, 32, 33]. Along similar lines, a good response was correlated with low levels of Th17 and was shown to be controlled by regulatory T cells (Tregs) in patients treated with ADA, not in those treated with ETA [34]. Th1 compartments were also reported to be increased in patients not responding to IFX [27] and, conversely, in patients in remission in response to ADA [26].

Furthermore, all the anti-TNF drugs IFX, ADA, CZP, and ETA induce IL-17^+^CD4^+^ T cells expressing IL-10 in RA patients [35]. The induction of IL-10 in association with IL-17 by Th17 suggests a modulatory role of those cells, but this needs to be demonstrated.

In conclusion, Th17 and Th1 compartments are increased in response to TNF inhibitors but a definitive answer as to whether they are linked to good or poor responses is needed. This is likely to depend on Th CD4^+^ T-cell phenotyping techniques, on the biologic administered, and the methodology used.

STAT6, which is associated with the Th2 response, was also induced in T cells from patients treated with ADA [36]. This would suggest a role for ADA in modifying T-cell polarization. Modifications of macrophage polarization induced by anti-TNF (our unpublished data) could also lead to changes in T-cell polarization.

The development of paradoxical psoriasis as a side effect of anti-TNF (ETA, IFX, or ADA) treatment in RA patients has been observed. The mechanism has been shown to involve IFN-α produced by plasmacytoid dendritic cells whose maturation is inhibited by anti-TNF [37] and not to the emergence of Th17 cells during treatment as previously suggested [38]. Recently, a new population of CD4^+^ T cells, called T peripheral helper (Tph) cells, has been identified in the synovial membrane of RA patients using mass cytometry technology [39]. Tph cells are CD4^+^ T cells that express high levels of the checkpoint protein PD-1 and, contrary to T-follicular helper cells (Tfh), do not express CXCR5. Tph cells induce the differentiation of plasma cells through IL-21. The inhibition of Tph by anti-TNFs [33] may prevent the differentiation of plasmablasts [39].

## Anti-TNF treatments affect Tregs in RA

There are 2 types of CD4CD25 FoxP3-positive Tregs, inducible (iTregs) and natural (nTregs). Inducible Tregs depend on TNF-R2 as exemplified by the observation that TNF-R2 is critical for stabilization and homeostasis of Tregs [40]. TNF has been reported to be either an activator or inhibitor of Tregs depending on the study, as reviewed in [41]. TNF was reported to inhibit both the phosphorylation of FoxP3 and the development of Tregs in correlation with an increase in IL-17- and IFN-γ-producing CD4^+^ T cells [42]. However, it was shown that Tregs did not lose their suppressive activity in the presence of TNF. Because TNF has costimulatory effects [18], T-effector cells (Teff) may appear resistant to the effect of Tregs [43], and this may have led to previous misinterpretation of the negative role of TNF on Tregs. It was first shown that Tregs from RA patients are present but defective and their function can be restored by IFX treatment. An induced population of iTregs, whose activity is mediated through IL-10 and TGF-β, is restored under the action of IFX, whereas defective nTregs are not [44]. This can be explained by a new mechanism of action with binding of ADA to tmTNF, which is strongly expressed by monocytes from RA patients. ADA induces higher levels of tmTNF in those monocytes and promotes interaction with TNF-R2-expressing iTregs, which subsequently expand [45]. Such a phenomenon is not observed with ETA. Thus, anti-TNF antibody, but not soluble receptor, induces iTregs through increased expression of tmTNF.

On the T lymphocyte side, the role of soluble versus tmTNF has been explored in several models. T lymphocyte-monocyte contact is important in inflammation. This involves, in part, tmTNF interaction with TNF-R2 on adjacent cells [46]. Blocking tmTNF on T lymphocytes impairs the production of TNF by monocytes [46], and tmTNF expressed by T cells is responsible for the modulation of IL-10 production by monocytes [47].

In T cells, IFX but not ETA induces IL-10 production through reverse signaling, showing disparity in the efficacy of biologics regarding molecular mechanisms [23] but suggesting a possible regulatory role for reverse signaling depending on the biologic used.

## Consequences of anti-TNF treatments on T-cell control of infections

Reactivation of tuberculosis during anti-TNF therapy by monoclonal antibodies and, to a lesser extent, by ETA has been a major drawback of biotherapies of rheumatic diseases [48]. Production of IFN-γ is, along with TNF, a major element of the T-cell immune response against tuberculosis. Nowadays, recommendations are to test for prior tuberculosis infection before anti-TNF treatments using interferon-gamma release assays (IGRAs) that detect specific T-cell response. Antituberculosis antibiotic prophylaxis has considerably reduced the risks of reactivation.

IFX triggers a reduction in CD8^+^ terminally differentiated effector memory CD45RA^+^ T cells (TEMRA cells) with antimicrobial activity against mycobacterium tuberculosis and is responsible for impairing the T-cell defense against microbes [49].

CD4^+^ T-cell proliferation and IFN-γ production against tuberculosis PPD and CFP-10 antigens were shown to be impaired by a 14-week treatment with anti-TNF in patients with a positive test for prior tuberculosis infection [50]. The inhibition was more pronounced in vitro with antibodies than with ETA.

CD8^+^-derived TNF is essential for antilisteria activity in mice. Patients treated with IFX are at higher risk for infections with listeria, another intracellular bacteria, than those treated with ETA [51].

Viral infections are controlled at least in part by CD4^+^ and CD8^+^ T lymphocytes through their cytotoxic activity and their release of cytokines such as TNF and IFN-γ [52]. Anti-TNF biotherapies have been shown to induce disparate changes in the antivirus immunity which may be due to modifications of SLO and/or direct inhibition of the antiviral effect of TNF. There is no clear evidence for a risk of varicella zoster virus (VZV) and cytomegalovirus (CMV) reactivation in patients undertaking biotherapies [53] and we have shown that the anti-CMV CD4^+^ response in RA patients treated with IFX, ADA, or ETA is conserved [54]. However, caution is required with respect to the safety of anti-TNF in patients with those viral infections.

Hepatitis B infections are controlled by T lymphocytes. Depletion of the T-cell response by anti-TNF treatments may explain the resurgence of hepatitis B chronic infections which may occur more frequently with antibodies than with ETA [55]. There is an increased risk of viral reactivation in patients with chronic HBV. Antiviral prophylaxis is required in these patients. It is not known whether the risks with different anti-TNFs are similar or not. Patients with past infection have no particular risk [56].

Recommendations with regard to hepatitis B and C infections in patients treated with anti-TNF have been proposed [57]. Caution is required in patients treated with anti-TNF regarding the follow-up of chronic infection or active infection. In any case, TNF inhibitors can be discontinued as they do not induce irreversible inhibition of TNF production [58].

## Consequences of anti-TNF on T-dependent B-cell responses

TNF is involved in T cell-dependent B-cell responses. Resting memory CD45RO^+^ T cells activated by cytokines, among them TNF, can provide help to B cells for the production of IgM, IgG, and IgA [59]. CD4^+^ T cells expressing tmTNF provide a costimulatory signal for B cells [60].

Regarding response to vaccines, clinical studies performed with influenza and pneumococcal [61] vaccination reported only modest decreases in antibody titers in patients treated with ADA and safe immunization. Vaccination recommendations for the physician are provided in a recent article [62].

A proportion of RA patients treated with anti-TNF biologics develop antidrug antibodies that can hamper the efficiency of treatments [63]. Due to its structure, ETA has lower immunogenicity than anti-TNF antibodies and anti-ETA antibodies seem to be non-neutralizing [63]. Antigen presenting cells take up anti-TNF antibodies as antigens and present epitopes to CD4^+^ T cells. Such immunogenicity of anti-TNF antibodies in RA patients suggests that there is no profound decay of T cell-mediated B-cell immunity. Thus, from a functional point of view, only a partial decrease of the B-cell response is observed in RA patients treated with anti-TNF. Although cumbersome, a way of reducing the immunogenicity of anti-TNF antibodies would be to identify T cell epitopes and to modify them accordingly. From a clinical point of view, prescribing anti-TNF with methotrexate, an immunosuppressive drug that reduces the production of Th1 cytokines [64], decreases the risk of antidrug antibodies.

## Conclusion

**Fig. 1:**
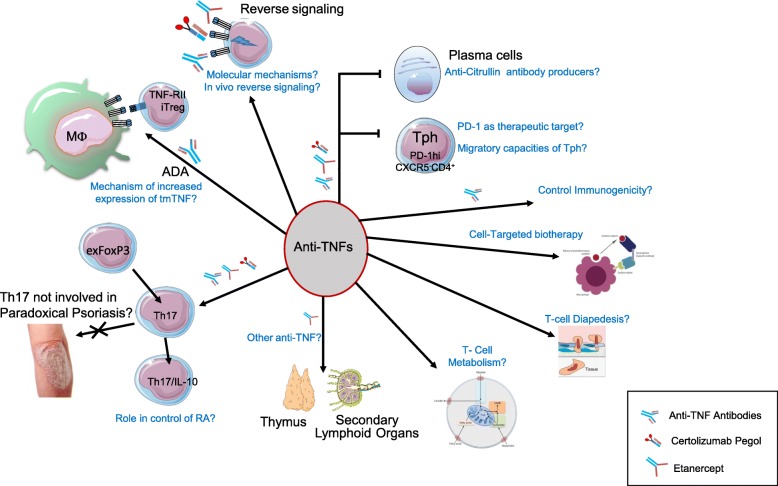
Summary of anti-TNF impact on T cells in RA and possible topics of interest for future investigations. Targets of antitumor necrosis factor (TNF) presented in this figure are developed in the main text. Questions raised, and possible topics of future research, are indicated: What is the mechanism of the increase of transmembrane (tm)TNF expression on macrophages that leads to expansion of inducible regulatory T cells (iTregs)? Are T helper (Th)17 cells definitely not responsible for paradoxical psoriasis? What is the role of interleukin (IL)-17/IL-10 producing T cells in the control of rheumatoid arthritis (RA)? Are anti-TNFs other than ETA modifying maturation of thymus and SLO? Are anti-TNFs modifying T-cell metabolism? Are anti-TNFs modifying T-cell diapedesis? What are the molecular mechanisms of reverse signaling? Is there a significant role for in-vivo reverse signaling? Is PD-1 a therapeutic target in RA? Do T peripheral helper (Tph) cells have specific migratory properties? Are plasmablasts induced by Tph pathogenic? Do they produce anti-CCP antibodies? How to modulate immunization against anti-TNF? How to improve targeted anti-TNF biotherapy?

Figure 1 summarizes the consequences of anti-TNF on T-cell homeostasis. Anti-TNF can regulate the T-cell responses in many ways. By inducing iTregs through TNF-RII and restoring T-cell function, these biologics contribute to reducing the autoimmune process. Although apparently contradictory, the induction of iTregs and the restoration of T-cell effector functions suggest that anti-TNF acts on multiple aspects of T-cell homeostasis. Although T cell-dependent B-cell activation is decreased, the risks of immunization resulting in anti-antibodies hampers the efficiency of treatment. In this regard, ETA induces less antidrug antibodies.

Differences between antibodies (IFX and ADA), and monovalent CZP, and ETA were outlined in the present review. They are summarized in Table 1. It appears that ETA and CZP induce less cell death and apoptosis than IFX and ADA. Alteration of the immune response to infections is less pronounced with ETA than with IFX and ADA but control of bacterial and viral infections is decreased by anti-TNF, and assessment of the infection and vaccine status is required. Vaccinations are recommended but not those using attenuated viruses or bacteria.

**Table 1:** Summary of specific effects of TNF inhibitors on T cells

	IFX	ADA	CZP	ETA
SLO	–	–	–	Patients lack germinal center development in tonsils [13]
Th1	in nonresponders [27]	in responders [26]	–	in responders [26]
Th17	in nonresponders [27]	in nonresponders [32]	–	in nonresponders [27, 32]
	–	Associated with ultrasound improvement [30]	–	Associated with ultrasound improvement [30]
	–	Good response correlated with low levels of Th17 [34]	-	No correlation of good response with low levels of Th17 [34]
	Induction of IL-17^+^ IL-10^+^ CD4^+^ T cells [35]	Induction of IL-17^+^ IL-10^+^ CD4^+^ T cells [35]	Induction of IL-17+ IL-10+ CD4+ T-cells [35]	Induction of IL-17^+^ IL-10^+^ CD4^+^ T cells [35]
Tph	Decrease in Tph [39]	–	Decrease in Tph [39]	Decrease in Tph [39]
Treg	Restoration of functional Tregs [44]	Expansion of iTregs through tmTNF-Mo/TNF-RII T-cell interaction [45]	–	No expansion of iTregs [45]
T-cell activation	Induction of STAT4 and STAT6 [36]	–	–	–
Reverse signaling	Induction of IL-10 in tm-Jurkat cells [23]	–	–	No induction of IL-10 in tm-Jurkat cells [23]
	Suppression of tm-Jurkat cell proliferation [23]	–	–	No suppression of tm-Jurkat cell proliferation [23]
	JNK activation in tm-Jurkat [23]	–	–	No JNK activation in tm-Jurkat [23]
Metabolism	Not affected [21]	–	–	–
Infections	Tb reactivation [48]	Tb reactivation [48]	Tb reactivation [48]	Lower rate of Tb reactivation than with Abs [48]
	Reduction of Tb-specific CD8^+^ memory cells [49]	–	–	–
	Inhibition of CD4^+^ response [50]	Inhibition of CD4^+^ response [50]	–	Inhibition of CD4^+^ response less pronounced than with Abs [50]
	Risk of listeria infection [51]	–	–	Lower risk of listeria infection than with sIFX [51]
	CD4^+^ response to CMV Ags conserved [54]	CD4^+^ response to CMV Ags conserved [54]	–	CD4^+^ response to CMV Ags conserved [54]
	Reactivation of HBV chronic infection [55]	Reactivation of HBV chronic infection [55]	–	Possibly less reactivation of HBV chronic infection [55]
Vaccination	Inadvertent vaccination with live vaccines (yellow fever, VZV) suggest they may be safer than expected [62]	Inadvertent vaccination with live vaccines (yellow fever, VZV) suggest they may be safer than expected [62]	Inadvertent vaccination with live vaccines (yellow fever, VZV) suggest they may be safer than expected [62]	Inadvertent vaccination with live vaccines (yellow fever, VZV) suggest they may be safer than expected [62]
	Pneumococcal and influenza vaccine immunogenicity not reduced by anti-TNF [61, 62]	Pneumococcal and influenza vaccine immunogenicity not reduced by anti-TNF [61, 62]	Pneumococcal and influenza vaccine immunogenicity not reduced by anti-TNF [61, 62]	Pneumococcal and influenza vaccine immunogenicity not reduced by anti-TNF [61, 62]
	No specific effect of TNF inhibitors on HBV protective immunity [56]	No specific effect of TNF inhibitors on HBV protective immunity [56]	–	No specific effect of TNF inhibitors on HBV protective immunity [56]
Antidrug antibodies	A proportion of patients develop antidrug antibodies	A proportion of patients develop antidrug antibodies	A proportion of patients develop antidrug antibodies	Fewer patients develop antidrug antibodies which appear to be less neutralizing
Cell death	Induction of ADCC and CDC in tm-Jurkat [24]	Induction of ADCC and CDC in tm-Jurkat [24]	No induction of ADCC and CDC in tm-Jurkat [24]	Lower induction of ADCC or CDC in tm-Jurkat in tm-Jurkat [24]
	Loss of cell viability of tm-Jurkat [24]	Loss of cell viability of tm-Jurkat [24]	No loss of cell viability of tm-Jurkat [24]	No loss of cell viability of tm-Jurkat [24]
Apoptosis	Apoptosis of tm-Jurkat [24]	Apoptosis of tm-Jurkat [24]	No apoptosis of tm-Jurkat [24]	No apoptosis of tm-Jurkat [24]
	Apoptosis of CD3-activated T cells [66]	Apoptosis of CD3-activated T cells [66]	No apoptosis of CD3-activated T cells [66]	Apoptosis of CD3-activated T cells [66]
	Spontaneous in-vitro apoptosis of CD4^+^CD25^+^ T cells diminished [25]	–	–	–

Only references in which modifications of Th1/Th17 are correlated with clinical response are listed

tm-TNF Jurkat is a model of Jurkat T cells transfected with a noncleavable form of TNF [23]

Golimumab is not listed because too few data were available on this biologic

– not available, *Ab* antibody, *ADA* adalimumab, *ADCC* antibody-dependent cell-mediated cytotoxicity, *Ag* antigen, *CDC* cell-dependent cytotoxicity, *CMV* cytomegalovirus, *CZP* certolizumab pegol, *ETA* etanercept, *HBV* hepatitis B virus, *IFX* infliximab, *IL* interleukin, *iTreg* inducible regulatory T cell, *s* soluble, *SLO* secondary lymphoid organs, *Tb* tuberculosis, *Th* T helper, *tm* transmembrane, *TNF* tumor necrosis factor, *Tph* T peripheral helper, *Treg* regulatory T cell, *VZV* varicella zoster virus

The mode of action, especially on T cells, of TNF-inhibitors is still not completely understood. For example, reverse signaling induced by TNF inhibitors must be explored in more detail. Future therapeutic strategy for RA should still take TNF inhibitors into account despite the availability of other biologics targeting other cytokines such as IL-6 and the more recent advent of JAKi. The choice of molecule should depend on better knowledge of the mode of action of the various TNF inhibitors. Nonspecific effects of anti-TNF antibodies on the immune system plead for a more targeted action such as bispecific antibodies targeting cells on the one hand and proinflammatory cytokine on the other [65].

## Abbreviations

ADA: Adalimumab
CMV: Cytomegalovirus
CZP: Certolizumab pegol
DC: Dendritic cell
ETA: Etanercept
IFX: Infliximab
IFN: Interferon
IL: Interleukin
iTreg: Inducible regulatory T cell
RA: Rheumatoid arthritis
SLO: Secondary lymphoid organs
Th: T helper
TGF: Transforming growth factor
tm: Transmembrane
TNF: Tumor necrosis factor
Tph: T peripheral helper
Treg: Regulatory T cell
T_RM_: Resident memory T cells
VZV: Varicella zoster virus
